# ‘Squeezing’ near-field thermal emission for ultra-efficient high-power thermophotovoltaic conversion

**DOI:** 10.1038/srep28472

**Published:** 2016-07-01

**Authors:** Aristeidis Karalis, J. D. Joannopoulos

**Affiliations:** 1 Research Laboratory of Electronics, Massachusetts Institute of Technology, Cambridge, MA 02139, USA; 2 Department of Physics, Massachusetts Institute of Technology, Cambridge, MA 02139, USA

## Abstract

We numerically demonstrate near-field planar ThermoPhotoVoltaic systems with very high efficiency and output power, at large vacuum gaps. Example performances include: at 1200 °*K* emitter temperature, output power density 2 *W*/*cm*^2^ with ~47% efficiency at 300 *nm* vacuum gap; at 2100 °*K*, 24 *W*/*cm*^2^ with ~57% efficiency at 200 *nm* gap; and, at 3000 °*K*, 115 *W*/*cm*^2^ with ~61% efficiency at 140 *nm* gap. Key to this striking performance is a novel photonic design forcing the emitter and cell single modes to cros resonantly couple and impedance-match just above the semiconductor bandgap, creating there a ‘squeezed’ narrowband near-field emission spectrum. Specifically, we employ surface-plasmon-polariton thermal emitters and silver-backed semiconductor-thin-film photovoltaic cells. The emitter planar plasmonic nature allows for high-power and stable high-temperature operation. Our simulations include modeling of free-carrier absorption in both cell electrodes and temperature dependence of the emitter properties. At high temperatures, the efficiency enhancement via resonant mode cross-coupling and matching can be extended to even higher power, by appropriately patterning the silver back electrode to enforce also an absorber effective surface-plasmon-polariton mode. Our proposed designs can therefore lead the way for mass-producible and low-cost ThermoPhotoVoltaic micro-generators and solar cells.

ThermoPhotoVoltaics (TPV)^1^,^2^,^3^,^4^ is a heat-to-electricity conversion mechanism, wherein Thermal radiation is absorbed by a semiconductor PhotoVoltaic (PV) cell. It is very favorable, as it involves no moving parts, allowing the possibility for compact, light (thus portable), quiet and long-lived generators, powerable from numerous sources, such as high-energy-density hydrocarbon^5^ or nuclear^6^ fuels, or solar irradiation^7^,^8^,^9^. Like any heat engine, a TPV system has the Carnot efficiency limit, which can only be achieved with monochromatic radiation matched to the semiconductor electronic bandgap. Absorbed thermal radiation below the bandgap is completely lost and far above it suffers thermalization losses. Reaching this limit in practical implementations has been challenging^4^.

The currently most developed TPV systems use the emitter far-field radiation to transfer thermal energy across a *mm*-scale vacuum gap to the PV cell, so their output power density is limited by the blackbody radiation limit. To get high efficiency, several methods have been proposed: selective narrowband (thus very-low-power)^10^,^11^,^12^,^13^,^14^ or wideband (so more thermalization loss)^15^,^16^,^17^ emitters, whose selectivity is though smeared significantly at high temperatures^16^; reflectors, to circulate below-bandgap photons back to the emitter, either on the PV-cell front surface, implemented by expensive filters^18^,^19^,^20^, or on its back-surface, but only if the substrate has low free-carrier absorption^21^; expensive tandem PV cells^21^.

In near-field TPV systems, thermal-energy transfer also via the evanescent modes can lead to significantly increased output power^22^,^23^,^24^,^25^. Systems with emitters employing a Surface Plasmon Polariton (SPP) resonance, tuned above the bandgap of a thick PV cell, spaced across a tiny (few-*nm*) vacuum gap, without^26^,^27^,^28^,^29^ and with^25^,^30^ a metal back-surface reflector, have been shown to indeed have increased power and efficiency. Systems with metal-backed thin-film semiconductor emitter and absorber, supporting coupled photonic resonances, were recently shown to exhibit high efficiency even at larger (100 *nm*) gaps^31^. However, as we show here, both these systems suffer in certain operating regimes from absorption losses by the necessarily-many free carriers in the semiconductor(s), an effect that has not been previously examined carefully. Semiconductor emitters are also limited by their relatively low melting temperatures and their bandgap shift and smearing at high temperatures^32^.

In this work, we propose a planar TPV system and a key design method to accomplish ‘squeezed’ narrowband near-field thermal-power transmission, with record-high heat-to-electricity efficiencies, at variably-high power levels, from low up to extremely-high emitter temperatures, with realistic material parameters and a large (practically realizable) vacuum gap. It builds upon the notion of impedance matching of coupled resonances that we recently introduced for TPV^33^, by enforcing it to the *crossed* resonant modes of a SPP emitter and a thin-film PV-cell absorber just above its semiconductor bandgap. The analysis takes into account free-carrier absorption in both PV-cell electrodes and temperature-dependent emitter properties. Similar material systems have been studied in the past (surface-phonon emitter^34^ and tungsten emitter^31^ with thin-film absorber), however, those systems were not designed with the above principles, which is why their reported efficiencies^31^ were significantly lower than those reported here.

## Results

### Photonic design and proposed structure

In a TPV system, an emitter *e*, at a high temperature *T*_*e*_, emits photons, some of which are absorbed by a semiconductor-diode PV-cell absorber *a*, at *T*_*a*_ < *T*_*e*_, which then converts them to a voltage *V* across itself and thus a current *I* into an externally connected load *l*. Other lossy objects or mechanisms in the system comprise the lossy background *b*. The Carnot efficiency is reached, for an ideal TPV system of no background losses (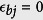) and monochromatic thermal transmission [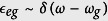], at *qV*/*E*_*g*_ = *η*_Carnot_ = 1 − *T*_*a*_/*T*_*e*_, where 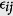 the *thermal transmissivity* between objects *i*, *j* (defined in Methods) and *E*_*g*_ = 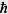
*ω*_*g*_ the energy of the semiconductor electronic band*g*ap. Therefore, to approach this limit for a real planar layered system (uniform in *xy*), the emitter *thermal emissivity* 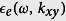 (also defined in Methods) should be designed close to 1 (its maximum per polarization) for a small frequency bandwidth just above the semiconductor bandgap and for as many in-plane wavevectors *k*_*xy*_ as possible, and close to 0 everywhere else. Significant obstacles usually are a large number of photonic modes outside the favorable frequency zone, the large broadening of the emitter modes at elevated temperatures, and the free-carrier absorption in the necessary PV-cell conducting electrodes.

Our design principle is to make both the planar emitter and absorber each support, near and below the bandgap, only one photonic mode, such that these two modes are substantially different (in wavevector *k*_*xy*_) at all frequencies, except right above the bandgap, where they cross and couple appropriately to achieve impedance matching. The selective property of coupled impedance-matched resonances means that 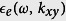 is high only near the resonant-crossing frequency, but low elsewhere. Mathematically, at the crossing wavevector, it has a double-Lorentzian frequency spectrum [Equation (11) of ref. 33], which can have very sharp transitions compared to the single-resonance Lorentzian [Equation (4) of ref. 33], even if the emitter resonance is very broad, thus effectively ‘squeezing’ its emission spectrum, as desired. Physically, impedance matching requires a large vacuum gap, inside which the evanescent tails of the two photonic modes overlap sufficiently to couple at resonance, while the penetration of the emitter-mode tail inside the absorber (and vice versa) is very weak, so transmission is low away from resonance. The large (>100 *nm*) gap is itself a huge advantage of a resonant TPV system, as fabricating few-*nm* gaps has been a main bottleneck of near-field TPV.

**Figure 1:**
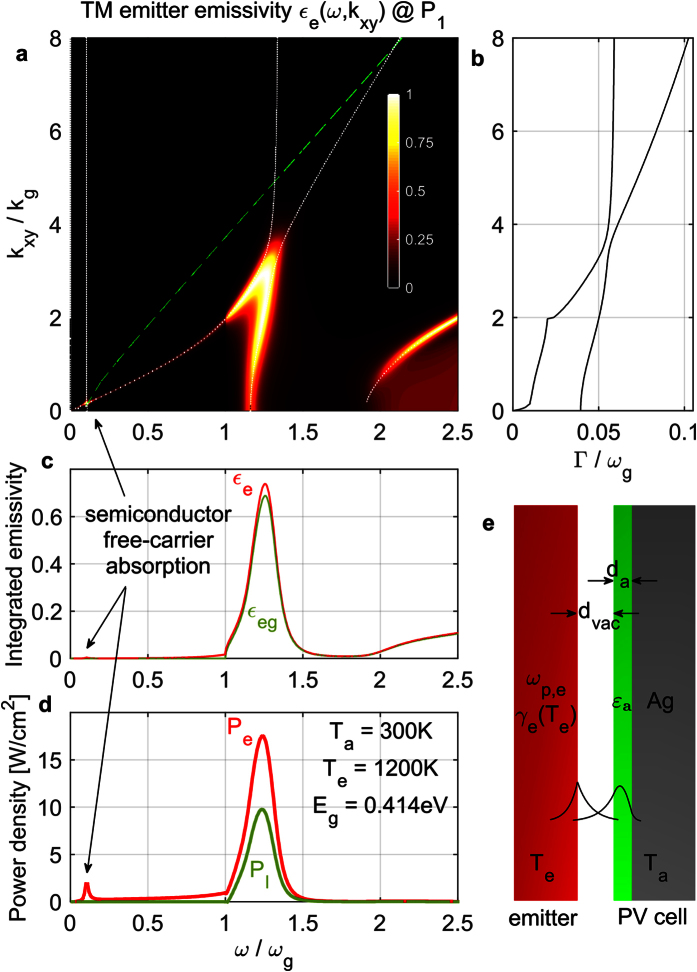
Results for optimized structure of Fig. 1e, at *T*_*e*_ = 1200 °*K*, *T*_*a*_ = 300 °*K* and with *E*_*g*_ = 4*k*_*B*_*T*_*e*_ = 0.414 *eV*. (**a**) TM emitter emissivity 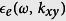 (color plot) and dispersion of system modes (dotted white lines); dashed green line is the semiconductor-material radiation cone. (**b**) Loss rates of the two system modes. Note the ‘kink’ in one mode loss-rate due to the onset of semiconductor inter-band absorption. (**c**) TM emitter emissivity 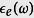 (red line) and emitter-bandgap transmissivity 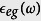 (green line) integrated over *k_xy_*. (**d**) TM emitter power *P_e_*(*ω*) (red line) and load power *P_l_*(*ω*) (green line) densities at the optimal-efficiency load voltage. (**e**) Proposed TPV structure of a plasmonic emitter and a silver-backed semiconductor thin-film absorber. The semiconductor *ε_a_* includes free carriers to model the front electrode. The coupled emitter-SPP and absorber-waveguide modal energy profiles are shown qualitatively.

A resonant crossing can be accomplished by photonic modes that, at the same frequency, have substantially different group velocities. The absorber semiconductor typically has a large relative permittivity (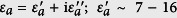), so a thin film will support tightly-confined waveguide modes, with positive group velocity of order 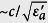. The modal confinement on the thin-film back side can be achieved via a metal, which can also serve as a back electrode for the PV cell or a Lateral Conduction Layer in Monolithic Interconnected Modules^21^ and, as we will see, removes modes from the radiation cone. To get a clear mode-crossing, the emitter photonic mode should have group velocity close to zero or even negative. Modes that exhibit such dispersion are SPPs: TM-polarized surface states on the interfaces of plasmonic and dielectric materials, like the vacuum gap^35^. The proposed structure, along with the typical energy-density profiles of the emitter and absorber modes, is shown in Fig. 1e.

The PV cell requires for operation two conductive electrodes, across which the output voltage *V* is induced and the load is connected. As described already, the back electrode can be provided by a metal. The front electrode must allow the hot photons to go through and be absorbed in the semiconductor. One implementation is by very highly doping a portion of the thin-film semiconductor, a process often called diffusion of the pn-junction ‘emitter’. The higher the doping concentration and the thickness of this front-electrode region, the lower its square resistance, but the higher the free-carrier absorption losses in it and the faster the radiative recombination of excited minority carriers before they diffuse towards the pn-junction depletion region to contribute to current. Contrariwise, free-carrier absorption and radiative recombination will be negligible in the depletion region, whose thickness decreases as the doping increases^32^. In a typical proposed PV cell, the semiconductor film will be so thin that it needs to be excessively doped to fit both the electrode and depletion regions inside it. Therefore, free-carrier absorption in the semiconductor may highly impact TPV efficiency and has to be modeled appropriately.

### Materials selection

The optimal choice of semiconductor bandgap *E*_*g*_ = 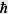
*ω*_*g*_ relates to the operating emitter temperature *T*_*e*_. The short-circuit net emitter power *P*_*e*,*sc*_ is given by Equation (3) in Methods with *V* = 0. If we ignore the mean-photon-number term 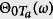, for high enough *T*_*e*_ ≫ *T*_*a*_, and normalize the integration variables by *u* = *ω*/*ω*_*g*_ and *v* = *k*_*xy*_/*k*_*g*_ (where *k*_*g*_ = *ω*_*g*_/*c* = 2*π*/*λ*_*g*_), then


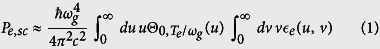


Since, by design, the narrowband emissivity will be high close to *u* = 1 and as small as possible at other frequencies, we can estimate the slowly-varying term 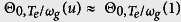, therefore 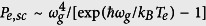 is roughly maximized, when *E*_*g*_ = 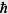
*ω*_*g*_ ≈ 4*k*_*B*_*T*_*e*_. As a guide, an emitter at 1200 °*K* optimally requires an absorber bandgap ≈0.4 *eV*, and, at 3000 °*K*, ≈1 *eV*. In all designs, we use *E*_*g*_ = 4*k*_*B*_*T*_*e*_, although different bandgap energies can also be used to improve TPV efficiency with the currently proposed method, with likely reduced power and efficiency. Many semiconductors fall inside this spectrum of bandgaps, like the commonly used GaInAsSb or GaInPAs quaternaries, and even silicon Si (1.1 *eV*) can be used for very high *T*_*e*_. A consequence of choosing *E*_*g*_ = 4*k*_*B*_*T*_*e*_ is that the emitter power scales as 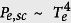, similarly to the Stefan-Boltzmann law for far-field thermal radiation.

To minimize the absorber-mode loss due to its penetration into the back-electrode metal, this should be the least lossy possible, so we use silver Ag.

**Figure 2:**
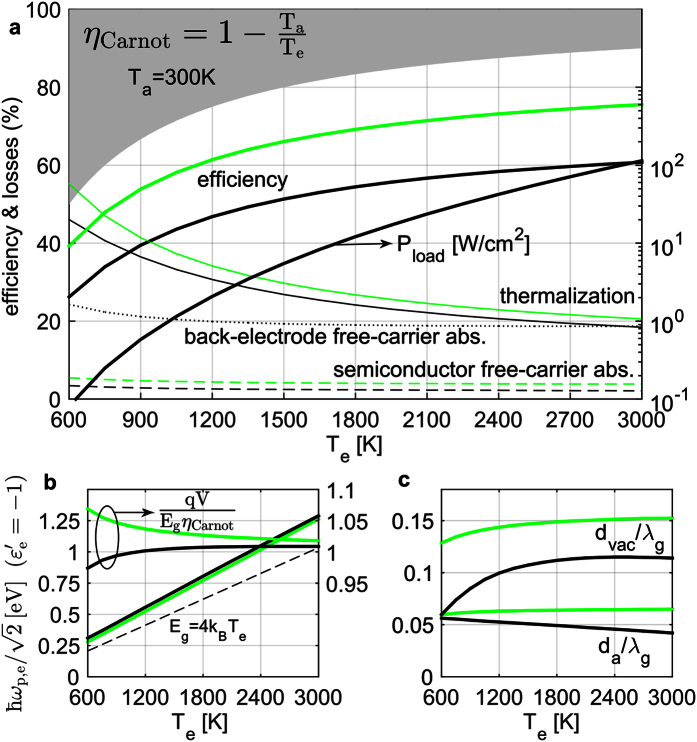
Optimization results vs emitter temperature *T*_*e*_. Ag back-electrode (black lines), PEC back-electrode (green lines). (**a**) Left axis: Efficiency (thick solid lines), thermalization losses (thin solid lines), semiconductor free-carrier absorption losses (dashed lines) and Ag back-electrode losses (dotted line); grey region is the Carnot limit on efficiency. Right axis: Output load power density. (**b**) Left axis: Optimal emitter plasma-frequency (scaled to cutoff frequency of a SPP on interface with vacuum, to be compared with Table 1); dashed black line shows *E*_*g*_ = 4 *k*_*B*_*T*_*e*_, for guidance. Right axis: Optimal load voltage (normalized to *E*_*g*_ and the Carnot efficiency). (**c**) Optimal vacuum-gap width and semiconductor thin-film thickness (normalized to *λ*_*g*_).

The emitter plasmonic material must be chosen to support a SPP mode with a cutoff frequency *ω*_*c*,*e*_ slightly above the bandgap *ω*_*g*_, say *ω*_*c*,*e*_ ≈ 1.2 *ω*_*g*_. Since typically *ω*_*g*_ ∈ (0.2–1) *eV*/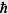, then *ω*_*c*,*e*_ ∈ (0.24–1.2)*eV*/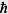. The SPP mode predominantly ‘sees’ the vacuum gap *ε*_*vac*_ = 1, so its cutoff frequency will occur, when Re{*ε*_*e*_(*ω*_*c*,*e*_)} ≈ −1 [from Equation (6)]. Therefore, we are looking for materials of high melting temperature, with *ε*′(*ω*) = −1 for *ω* roughly in (0.24–1.2)*eV*/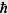. This range is confirmed by our simulations, as shown later in Fig. 2b. In Table 1, we show a list of such materials. In general, there are several refractory metals, metal carbides, nitrides^36^ and silicides that can be used for the emitter. ZrC and TiC seem two promising candidates, while the most common TPV emitter material, tungsten W, can also be used for very high *T*_*e*_ (perhaps even matched with a Si thin-film PV cell).

### Thermal emissivity spectrum for optimized structure

In order to evaluate the performance of the proposed mechanism for enhanced TPV efficiency, we perform optimization to calculate the maximum efficiency attainable. First, we consider the emitter at temperature *T*_*e*_ = 1200 °*K* and the PV cell at room temperature *T*_*a*_ = 300 °*K*, with *E*_*g*_ = 4*k*_*B*_*T*_*e*_ = 0.414 *eV*. For the structure of Fig. 1e, the optimization parameters are the emitter plasma frequency *ω*_*p*,*e*_, the vacuum-gap width *d*_*vac*_, and the absorber thickness *d*_*a*_.

In Fig. 1a, we show a color plot of the resulting TM emitter thermal emissivity spectrum 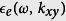 of the maximum-efficiency photonic structure, with its modes overlaid in dotted white lines. Note that the optimization was done considering both polarizations, however the TE contribution to the emissivity is comparatively small and is not shown in Fig. 1a, for clarity.

The resonant crossing (‘X-shape’) of the emitter SPP mode and the absorber first waveguide mode is clearly visible, and their coupling indicated by their splitting. Emissivity is maximized at 1 with a double-Lorentzian profile, exactly at the point of resonant crossing and coupling, slightly above the bandgap, and indeed the amount of the splitting ≈2*κ* is approximately satisfying the impedance matching condition 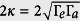 33, when compared to the loss rates Γ of the two system modes, shown in Fig. 1b.

Due to the metallic back electrode, the emitter and absorber modes cross-couple in a way that, of the two resulting system ‘super-modes’, the higher-frequency one has a (*k*_*xy*_ = 0) cutoff. The optimal absorber thickness *d*_*a*_ is such that this cutoff is slightly above the bandgap, where the emitter emissivity is then high for a large range of wavevectors, a very desirable feature to enhance output power and efficiency. The lower-frequency (no-cutoff) system ‘super-mode’ leads to some undesired below-bandgap emissivity, which stems primarily from coupling into losses of the silver back electrode. However, this electrode works overall beneficially, by essentially removing, from the radiation cone below the bandgap, many absorber modes, which would become additional lossy channels for the emitter to emit into.

One exception, an absorber below-bandgap mode not removed by the metal back electrode, is a SPP mode on the interface between the vacuum gap and the doped semiconductor, due to the free carriers of the latter inducing plasmonic-material behavior [with 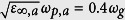, as seen in Equation (7)]. The upper-cutoff frequency for this SPP mode will be below the bandgap [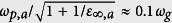, using Equation (6)], where this absorber free-carrier SPP also couples to the emitter SPP and there is an associated undesired emissivity peak. As we will see, this front-electrode loss mechanism can have a large impact on efficiency in other topologies or power levels.

The second TM mode of the thin-film absorber is also evident at higher frequencies and also the high emitter emissivity associated with this mode’s exponential tails reaching the emitter. This emissivity is also undesired, as it will be associated with large thermalization losses.

In Fig. 1c, we plot the emitter emissivity 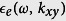 and the emitter-bandgap transmissivity 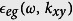 both integrated vs. *k*_*xy*_ [as required by Equations (3), (4)], and, in Fig. 1d, the emitter and load power density spectra [essentially the integrands of Equations (3), (4)]. In comparing Fig. 1c,d, multiplication by the exponentially decreasing mean-photon number 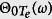 leads to a beneficial suppression of the high-frequency emissivity into the second absorber mode, but unfortunately ‘amplifies’ the below-bandgap losses associated with free-carrier absorption in the semiconductor and in the silver back electrode. However, as we will see later, in comparison to a bulk PV cell, the efficiency is substantially higher, because the double-Lorentzian spectrum profile and the relatively large vacuum gap have led in Fig. 1c to much suppressed free-carrier absorption loss to start with. The emitter near-field thermal power density spectrum (Fig. 1d) is almost a single impressively-narrowband peak, as desired for high efficiency and power TPV.

### Optimized performance and design vs. emitter temperature and vs. load power density

The optimization procedure is performed for *T*_*e*_ ∈ (600–3000) °*K*, using *E*_*g*_ = 4 *k*_*B*_*T*_*e*_. The resulting optimized efficiency vs. *T*_*e*_ is shown in Fig. 2a (thick black line) and is compared to the Carnot limit (grey region) and also to the case of a Perfect-Electric-Conductor (PEC) back electrode (thick green line). The system with the PEC back electrode lies 10–15% below *η*_Carnot_, while the one with a silver electrode only 25–30% below *η*_Carnot_. We also show the contributions of the different loss mechanisms: Thermalization losses (of order ~1 − *qV*/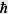
*ω* per absorbed photon, *qV* < *E*_*g*_ ≤ 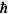
*ω* - thin solid lines) are the largest ones, the silver electrode losses (dotted black line) are around 20% and the free-carrier absorption losses (dashed lines) are suppressed due to the large vacuum gap to less than 4%. As temperature increases, the output load power density, also shown in Fig. 2a (thick black line – right axis), increases due to both the 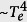 dependence and also the increase of efficiency. In Fig. 2b (left axis), we show the optimal emitter 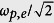, at which 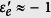, so that it can be compared to the materials in Table 1. 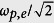 indicates the cutoff for an emitter-vacuum SPP and needs to lie above *ω*_*g*_, also shown for guidance (dashed black line). For a PEC back electrode and high enough *T*_*e*_, all normalized geometrical parameters (Fig. 2c) are fairly constant with *T*_*e*_, indicating that the optimal structure simply scales with *T*_*e*_. For a silver back electrode, the optimal *d*_*vac*_/*λ*_*g*_ is smaller than that for a PEC, since the additional silver losses induce a larger absorber-mode loss rate Γ_*a*_ and therefore the absorber needs to come closer to the emitter, for an increased coupling *κ* to achieve impedance matching. Furthermore, since the silver plasma frequency (4.65 *eV*/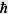) effectively decreases relatively to *ω*_*g*_ = 4*k*_*B*_*T*_*e*_/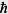, as *T*_*e*_ increases, the normalized PV-cell thickness *d*_*a*_/*λ*_*g*_ needs to be reduced, so that the absorber mode will remain at the same relative frequency above the increasing bandgap. The optimal load voltage *qV*/*E*_*g*_ (Fig. 2b right axis) follows pretty closely the value *η*_Carnot_ = 1 − *T*_*a*_/*T*_*e*_.

**Figure 3:**
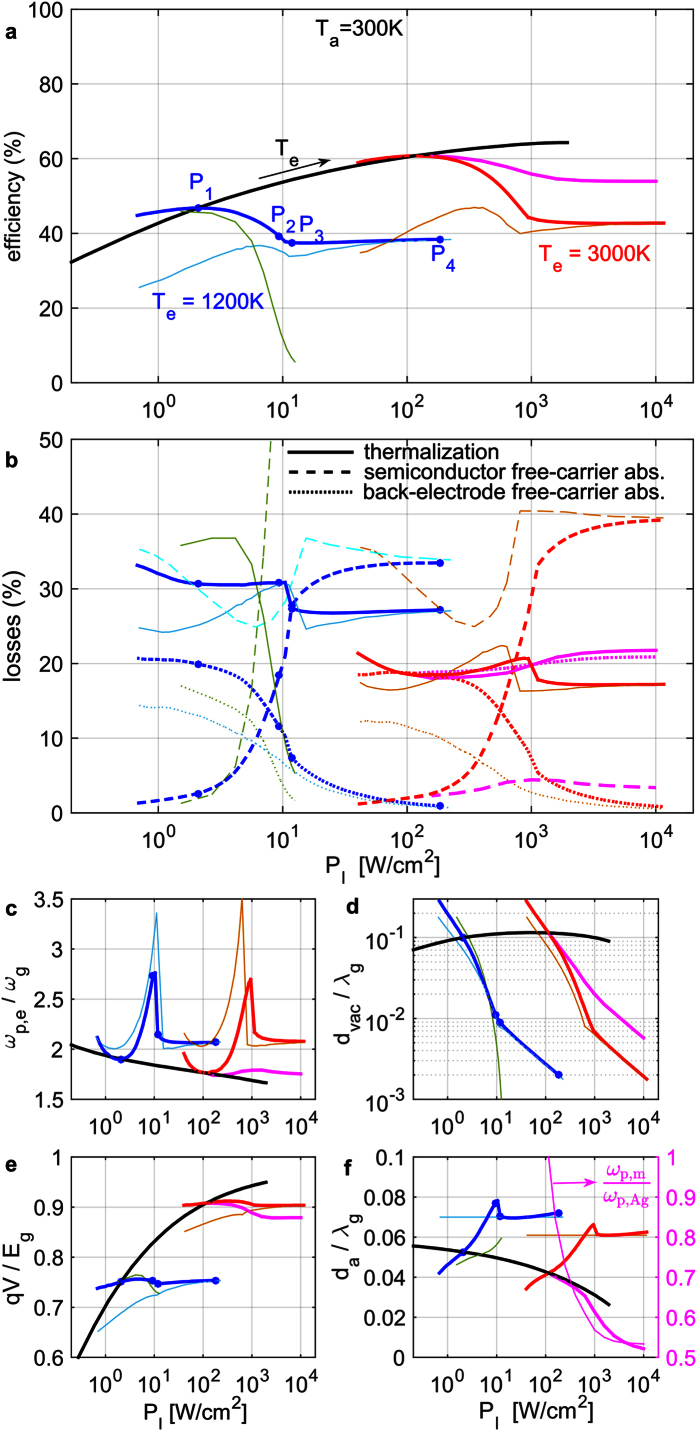
Optimization results vs output load power density *P*_*l*_. Figure 1e (blue lines at *T*_*e*_ = 1200 °*K*, red lines at 3000 °*K*), Fig. 5d (magenta lines at 3000 °*K*), Fig. S1a with *d*_*a*,base_ = 5*λ*_*g*_ (cyan lines at 1200 °*K*, orange lines at 3000 °*K*) and Fig. S2a (green lines at 1200 °*K*). Black lines are Fig. 2 results, parametrized by *T*_*e*_. (**a**) Efficiency. (**b**) Thermalization losses (solid lines), semiconductor free-carrier absorption losses (dashed lines) and back-electrode losses (dotted lines). (**c**) Optimal emitter plasma frequency. (**d**) Optimal vacuum-gap width. (**e**) Optimal load voltage. (**f**) Left axis: Optimal semiconductor thin-film thickness. Right magenta axis: Plasmonic back-electrode effective plasma frequency (normalized to Ag plasma frequency).

**Figure 4:**
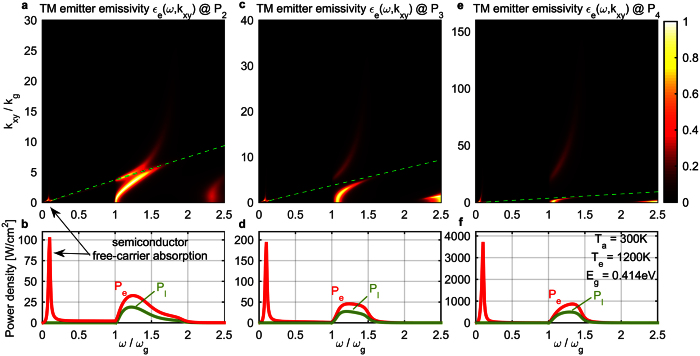
Spectra for optimized results of Fig. 3 blue lines (Fig. 1e system at *T*_*e*_ = 1200 °*K*) at 3 load-power levels indicated on Fig. 3 with blue dots: (**a,b**) *P*_2_, (**c,d**) *P*_3_, (**e,f**) *P*_4_; *P*_1_ spectra were shown in Fig. 1a,d. (**a,c,e**) TM emitter emissivity 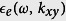 (color plot); green line is the semiconductor-material radiation cone. (**b,d,f**) TM emitter power *P*_*e*_(*ω*) (red line) and load power *P*_*l*_(*ω*) (green line) densities at the optimal-efficiency load voltage.

The performance of any electronic power converter, including a TPV one, is always ultimately judged by the efficiency achievable as a function of the power delivered to the load. Therefore, in Fig. 3 we replot vs. *P*_*l*_ the results of the previous optimization, with *T*_*e*_ being a parameter (thick black lines). Furthermore, we perform, at fixed *T*_*e*_ = 1200 °*K* (thick blue lines) and *T*_*e*_ = 3000 °*K* (thick red lines), a constrained optimization, with parameters same as before, only with the constraint that *P*_*l*_ takes prescribed values. As the desired output power increases, the necessary vacuum gap width decreases (Fig. 3d), as should be expected. Effectively, impedance matching and thus high emissivity must be achieved at a higher wavevector (and always at a frequency just above bandgap for good efficiency), so that the *k*_*xy*_ integration in Equation (3) will lead to more power. However, the absorber waveguide mode has an upper *k*_*xy*_-limit, the light-line of the semiconductor material (indicated in Figs 1a and 4a,c,e with dashed green lines). Therefore, resonant crossing and coupling of this absorber mode with the emitter SPP (and the approximately double-Lorentzian emissivity profile) is possible only up to approximately the power level *P*_2_ (Fig. 4a,b). Between *P*_1_ and *P*_2_, as this ‘good’ resonant coupling worsens, the vacuum gap decreases so much, that the ‘bad’ below-bandgap coupling of the emitter SPP to the absorber-free-carrier SPP increases significantly, leading to a large emissivity peak below the bandgap (Fig. 4b) and thus the associated losses are much larger (Fig. 3b) and efficiency drops (Fig. 3a). From that point on, the absorber needs to get so close to the emitter, that the emitter SPP has substantial energy inside the absorber, so it is more beneficial to redesign this single mode to get an efficient impedance-matched single-Loretzian emissivity profile^33^. So the optimal solution for *ω*_*p*,*e*_/*ω*_*g*_ (Fig. 3c) and *d*_*a*_/*λ*_*g*_ (Fig. 3f) has an abrupt change from *P*_2_ to *P*_3_. For even higher power, the emissivity is dominated by this tightly confined emitter-absorber SPP mode, whose dispersion moves to higher wavevectors, as the vacuum gap further decreases^35^. Its modal energy decays faster in the absorber and sees less the silver back electrode, whose associated losses then drop (Fig. 3b). In essence, at *P*_4_ (Fig. 4e,f) the system looks a lot like a plasmonic emitter separated by a tiny gap from a bulk semiconductor absorber^26^,^27^,^28^,^29^. *qV*/*E*_*g*_ (Fig. 3e) again stays close to *η*_Carnot_ for all power levels.

### Surface-Plasmon-Polariton absorber

There is another way, however, to achieve resonant coupling between emitter and absorber also at high power: if the absorber supports a SPP mode too. Note that this typically requires the semiconductor film to be even thinner.

**Figure 5:**
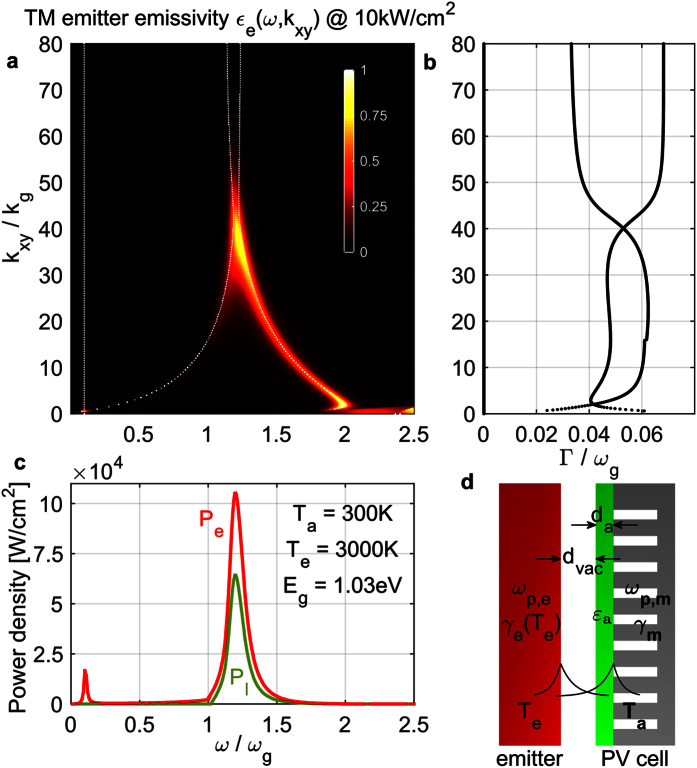
Results for optimized structure of Fig. 5d, at *T*_*e*_ = 3000 °*K*, *T*_*a*_ = 300 °*K*, *P*_*l*_ = 10 *kW*/*cm*^2^ and with *E*_*g*_ = 4 *k*_*B*_*T*_*e*_ = 1.03 *eV*. (**a**) TM emitter emissivity 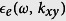 (color plot) and dispersion of system modes (dotted white lines). (**b**) Loss rates of the two system modes. Note the small ‘kink’ in one mode loss-rate due to the onset of semiconductor inter-band absorption. (**c**) TM emitter power *P*_*e*_(*ω*) (red line) and load power *P*_*l*_(*ω*) (green line) densities at the optimal-efficiency load voltage. (**d**) Proposed TPV structure of a plasmonic emitter and a semiconductor thin-film absorber backed by an effective plasmonic electrode via a dense periodic patterning of holes on silver. The semiconductor *ε*_*a*_ includes free carriers to model the front electrode. The coupled emitter-SPP and absorber-SPP modal energy profiles are shown qualitatively.

One such resonant absorber-SPP implementation is to make the back electrode of a plasmonic material (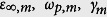) with a small enough *ω*_*p*,*m*_ that the SPP supported at its interface with the semiconductor^35^ has a cutoff (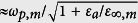) just above the bandgap *ω*_*g*_. Metallic materials with relatively small *ω*_*p*,*m*_ (e.g. those presented in Table 1) or very-highly doped semiconductors or highly doped conducting oxides, whose *ω*_*p*,*m*_ is tunable via the doping concentration, can be used as a plasmonic back electrode. However, we saw (Fig. 3b) that, even for low-loss silver, there are significant losses at the back electrode. These will be even worse for a SPP mode, which by construction relies on significant modal penetration into the plasmonic material, with a SPP-modal loss rate *γ*_*m*_/2 in the limit of large *k*_*xy*_^37^. Unfortunately, most of the above-mentioned materials typically have too large *γ*_*m*_ for this proposed mechanism to work efficiently. Instead, we suggest using silver metallo-dielectric photonic crystals, which are metals with periodic geometry patterning, that can exhibit effective plasmonic behavior, with plasma frequency lower than the used metal (silver) and tunable via the fill factor of the patterning^38^. In a sense, one gets a material, which is a (fill-factor weighted) average of the consisting metal and dielectric (often air/vacuum). The period of the geometric patterning has to be sufficiently smaller than the SPP propagation wavelength 2*π*/*k*_*xy*_, for the effective-medium approximation to hold. These (effective SPP) surface modes were later coined as ‘spoof’ plasmons for the subcase of a metal with a 2D periodic patterning of holes on its flat interface^39^. Such a back-electrode implementation for the currently proposed TPV systems is shown in Fig. 5d. *ω*_*p*,*m*_ cannot be much smaller than the silver value 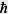
*ω*_*p*,*Ag*_ = 4.65 *eV*, so this scheme may be more applicable for very high emitter temperatures, for which the optimal semiconductor bandgap is high (~1 *eV*), thus we perform this optimization only for *T*_*e*_ = 3000 °*K*.

The results are shown in Fig. 3 with thick magenta lines, assuming silver losses are independent of patterning (see Methods). In Fig. 5a, we show a color plot of the resulting TM emitter thermal emissivity spectrum 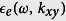 of the maximum-efficiency photonic structure at *P*_4_ = 10 *kW*/*cm*^2^, and the corresponding emitter/load power densities in Fig. 5c, exhibiting again the desired narrowband peak. The system modes’ dispersion is overlaid in Fig. 5a with dotted white lines and in Fig. 5b we plot the loss rates of the two coupled SPP modes, which can be confirmed as approximately impedance matched (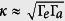). Clearly, in the case of patterning-independent silver losses, a large TPV efficiency could be achieved even for very high power levels (Fig. 3a). To get impedance matching of the coupled resonances (Fig. 5a) at higher power, and thus higher *k*_*xy*_, but close to *ω*_*g*_, a thinner semiconductor film and lower effective plasma frequency are required^35^ (Fig. 3f), and a vacuum gap larger than the non-resonant case (Fig. 3d). Therefore the semiconductor free-carrier losses stay low even for increasing power, and it is the back electrode losses that limit the performance (Fig. 3b), as predicted.

Note that another, truly-planar implementation of a resonant absorber-SPP using silver is possible, by placing, at the back side of the semiconductor film, an ultra-thin silver film on a substrate. Its thickness can be tuned to shift one SPP frequency down, close to 1 *eV*. However, simulations (not shown) suggest this method is less efficient, since such a SPP relies heavily on the lossy silver-film electrons for confinement and the frequency down-shift.

### Comparison to other topologies

It is instructive to compare our proposed near-field TPV systems to previously studied ones, when also optimized for efficiency, but including modeling of semiconductor free-carrier absorption, which has severe impact on efficiency and was omitted in previous studies (details in Supplementary information).

Consider the Fig. 1e topology, but with a bulk non-resonant semiconductor absorber (Fig. S1a)^26^,^27^,^28^,^29^,^30^. Its optimal performance is shown in Fig. 3 with cyan and orange lines for *T*_*e*_ = 1200 °*K* and 3000 °*K* respectively. Clearly, at low power, the efficiency is significantly lower than in the thin-film absorber case (Fig. 3a). Most of the losses come from free-carrier absorption within the semiconductor bulk (Fig. 3b), because this single-mode impedance matching requires a much smaller vacuum gap at the same power level (Fig. 3d) and thus the emitter SPP couples strongly with the absorber free carriers. At higher power (and wavevectors), the thin-film is also non-resonant, with the emitter SPP mode decaying fast into the absorber (Fig. 4e,f), so the two systems (thin-film vs. bulk) are electromagnetically similar and the silver back electrode does not impact efficiency greatly (Fig. 3b).

For a system of a tungsten-backed thin-film semiconductor emitter and a silver-backed thin-film matched-bandgap-semiconductor absorber (Fig. S2a)^31^, the optimal performance is shown in Fig. 3 with green lines for *T*_*e*_ = 1200 °*K* (for comparison, although too high for most semiconductors). Assuming single-mode operation for each resonant film, at low power, efficiency is similarly high as the plasmonic-emitter system, since the resonance benefits are present here too: high above-bandgap emissivity due to impedance-matched coupled resonances, suppressed free-carrier absorption due to large vacuum gap (Fig. 3d), removal of below-bandgap modes due to the metallic (W, Ag) reflectors. However, as the desired load power increases, the waveguide modes meet their *k*_*xy*_-limit and the necessary vacuum gap decreases (Fig. 3d), so emissivity related to the emitter and absorber free-carriers increases strongly (Fig. 3b), and efficiency drops very fast (Fig. 3a).

## Discussion

In a practical implementation of the proposed TPV structures, an ultrathin refractory oxide film, such as ThO_2_ (*T*_*melt*_ = 3660 °*K*), HfO_2_ (3031 °*K*) and ZrO_2_ (2988 °*K*), can be deposited on the plasmonic emitter to prevent its oxidization from the little oxygen potentially present in the vacuum gap.

The semiconductor absorption spectrum can also be modified to have a more selective response above its bandgap, via the use of quantum wells, wires or dots^40^. However, we do not expect significant improvement in performance, since free-carrier absorption in the electrodes is the main limiting factor and can only be mitigated via resonant photonic design, to increase the vacuum gap and reduce direct coupling between the emitter and absorber free electrons. This was confirmed by simulations using a step-function (quantum well) absorption profile.

In conclusion, we have demonstrated near-field TPV systems with very high efficiency and output power, using planar SPP thermal emitters and silver-backed thin-film PV cells, spaced by ample vacuum gaps. Using realistic material parameters and proper modeling of the front-electrode free-carrier absorption and the emitter heating, performance examples include: at *T*_*e*_ = 1200 °*K*, output 2 *W*/*cm*^2^ with ~47% efficiency (e.g. using ZrC emitter and 160 *nm*-thick InAs absorber at 300 *nm* vacuum gap), at *T*_*e*_ = 2100 °*K*, 24 *W*/*cm*^2^ with ~57% efficiency (e.g. using TiC emitter and 80 *nm*-thick GaSb absorber at 200 *nm* gap), and, at *T*_*e*_ = 3000 °*K*, 115 *W*/*cm*^2^ with ~61% efficiency (e.g. using W emitter and 50 *nm*-thick Ga_0.3_In_0.7_P_0.6_As_0.4_ absorber, grown on InP, at 140 *nm* gap). By appropriately patterning holes into the silver back-electrode, provided silver losses do not increase substantially, 1 *kW*/*cm*^2^ with ~56% efficiency and 24 *nm* gap may even be attainable. To our knowledge, such performance has never been predicted before for realistic TPV cells.

The actual performance of real TPV cells should differ only little from our predictions, with respect to the electronic details of the semiconductor pn-junction. It should depend more, though, on the desired front-electrode square resistance and the corresponding overall semiconductor doping level. However, our analysis shows that, for a given doping level, the currently proposed topologies and design perform better than previously suggested ones. Therefore, we believe this design can pave the way for useful mass-producible (and potentially low-cost) TPV micro-generators and solar TPV cells.

## Methods

### Calculational approach

Consider a planar system (uniform in *xy*) of multiple layers (stacked in *z*), where layer *j* is at a non-zero temperature *T*_*j*_ and potentially has a voltage *V*_*j*_ across it. At this thermal and chemical equilibrium, the excited random sources inside lossy layer *j* generate photons at every frequency *ω* with mean number 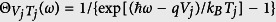
41. The thermal power density (per area *A*) transmitted from the emitting layer *j* to an absorbing *i*, with *T*_*i*_ = 0, can be written as^42^


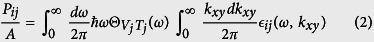


where *k*_*xy*_ is the in-plane wavevector and 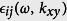 is defined as the *thermal transmissivity* from *j* to *i*. If the system is reciprocal, 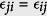. The net thermal power emitted from layer *j*, when all other *T*_*i* ≠ *j*_ = 0, is 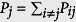 and leads to the definition of *thermal emissivity* 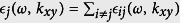, which is a generalization of the regular (wavevector-independent) emissivity^33^. The transmissivity 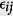 and emissivity 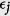 physically quantify how many photons emitted by layer *j* at mode (*ω*, *k*_*xy*_) are absorbed by respectively one (*i*) or all other (*i* ≠ *j*) layers (including the two semi-infinite boundary layers), and thus, from photon-number conservation, they both have maximum value 1 for each of the two decoupled (for isotropic media TE/TM) polarizations. They can be calculated semi-analytically, using an exact scattering-matrix method^42^ (details in Supplementary information).

In a TPV system, the thermally-excited random sources in the emitter *e* generate photons with mean number 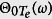, the sources related to the voltage-generating absorption mechanism *g* of the semiconductor absorber *a* (inter-band absorption across its band*g*ap) generate photons with mean 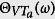, and those related to other loss mechanisms in the semiconductor (such as free-carrier, inter-valence-band, inter-valley-conduction-band absorption and non-radiative electron-hole recombination^43^) or in the other PV-cell background layers (labeled collectively by *b*) generate photons with mean 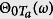. The system efficiency *η* is the ratio of the load power *P*_*l*_ = *V* ⋅ *I* over the net emitter power outflow *P*_*e*_ at all frequencies. To calculate *P*_*e*_, one uses superposition of all photons emitted by *e*, each with energy 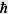
*ω*, minus those reabsorbed. Similarly, to calculate *I*, one superposes all photons absorbed by *g*, each leading to an electron-hole pair of charge *q*, minus those reemitted. Then, from Equation (2) for a planar geometry and using 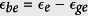, 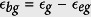 and reciprocity 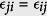:


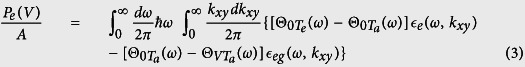



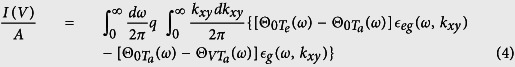


In contrast to regular PV systems, in TPV, both the emitter power *P*_*e*_ and load power *P*_*l*_ depend on the induced operating voltage *V*, settable via the load impedance *R* = *V*/*I*, thus *η* is maximized at a larger *V* than *P*_*l*_^33^. Note that *qV* < *E*_*g*_ will always hold, so that 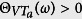 for all frequencies *ω* > *ω*_*g*_, at which the bandgap absorbs/emits (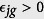). If *E*_*g*_ − *qV* ≫ *k*_*B*_*T*_*a*_, the Boltzmann approximation 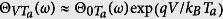 can explicitly extract the *V* dependence in Equations (3),(4), which are very general, containing several subcases (no emitter/simple diode, no absorber, ideal TPV)^33^. They are used for all results in this article, with *V* chosen to maximize efficiency. Our *η* definition does not include electrical losses due to (series and shunt) electrode resistances, which are thus incorporated in the load.

### Materials modeling

Throughout this article, we model free carriers, in the plasmonic material and in the PV-cell electrodes via the Drude model, namely the relative dielectric permittivity


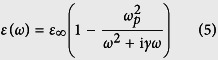


with plasma frequency 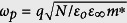 and loss factor 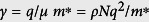, where *N* is the carrier density, m^*^ the effective mass of the carriers (electrons or holes), *μ* the carrier mobility and *ρ* = 1/*qNμ* the dc resistivity. At an interface with a dielectric of relative permittivity *ε*_*d*_, a SPP mode is supported with upper cutoff frequency (as *k*_*xy*_ → ∞) at


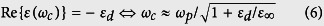


Our proposed mechanism improves performance irrespectfully of the precise electronic details of the pn junction in the semiconductor absorber (e.g. separate electrode and depletion regions). Thus it is sufficient to consider uniform ‘average’ dielectric properties, including both free-carrier and inter-band absorption. Surface recombination is assumed negligible via the use of passivation layers, but bulk radiative recombination is properly accounted for by the 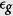 term in Equation (4) (see also Supplementary information). We use *ε*_∞,*a*_ = 14, appropriate for GaInAsSb semiconductors. For the free-carrier Drude term, we assume a carrier mobility scaling 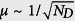, which is a simple but fair approximation for large ranges of the doping concentration (and thus free-carrier density) *N*_*D*_ in semiconductors. Then, as *ω*_*g*_ increases (and single-mode film thickness decreases, *d*_*a*_ ~ *λ*_*g*_) to maintain roughly constant electrode square resistance *R*_*sq*_ = *ρ*/*d*_*a*_, we use scaling 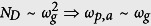. With typical GaInAsSb values for the electron and hole effective masses 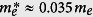 and 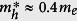, where *m*_*e*_ the electron mass, and typical carrier-density levels from previously reported TPV cells^30^,^44^, we use 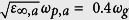. For example, this value corresponds to *N*_*e*_ ≈ 7 × 10^17^ *cm*^−3^ electrons or *N*_*h*_ ≈ 8 × 10^18^ *cm*^−3^ holes at *E*_*g*_ = 0.4 *eV* (*T*_*e*_ = 1200 °*K*). Furthermore, matching typical GaInAsSb mobility values^44^, we use 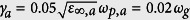. Inter-band absorption for direct-bandgap bulk semiconductors scales as 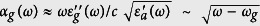, therefore 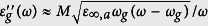, since 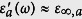 at *ω* > *ω*_*g*_. The dimensionless constant *M* depends on the conduction-band electron and valence-band hole effective masses, and is around 0.7 for GaInAsSb^29^,^44^. Therefore, we model the semiconductor absorber/electrode with dielectric permittivity


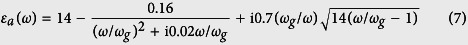


Silver in the back electrode is modeled with Drude parameters *ε*_∞,*Ag*_ = 4, 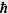
*ω*_*p*,*Ag*_ = 4.65 *eV* and 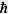
*γ*_*Ag*_ = 0.023 *eV*^45^,^46^,^47^.

For the plasmonic-emitter Drude modeling, in our design optimizations, we leave *ω*_*p*,*e*_ as an optimization variable and we assume *ε*_∞,*e*_ = 1, for simplicity. To quantify the loss factor *γ*_*e*_ of the hot emitter, we have to take into account how losses increase with temperature. Ref. 48 describes a model for the temperature dependence of resistivity, which should also hold for *γ*_*e*_, since *N* and m^*^ do not significantly depend on temperature for metallic-type materials. We use the simplified model


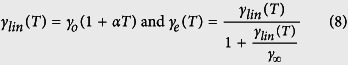


From parameters and measurements for ZrC and TiC from ref. 48, we deduce and use approximate values *γ*_*o*_ = 0.05 *ω*_*p*,*e*_, *α* = 0.002/°*K* and *γ*_∞_ = 2*γ*_*o*_. Note that, compared to silver with 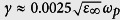, these are much lossier metallic-type materials and they become even lossier (almost twice) at very high temperatures. The associated broadening has been a key problem for prior TPV systems with metallic emitters, but, as discussed, a coupled-resonant system circumvents this issue.

The effective-plasmonic back electrode is modeled as a ‘tunable silver’ with *ε*_∞,*m*_ = 4 and *ω*_*p*,*m*_ being an additional optimization parameter, irrespectfully of the exact details of the geometric patterning that achieve it. To precisely model the loss factor *γ*_*m*_, one would need to know the geometry details. To get an estimate avoiding this complexity, we instead use the simple material-averaging argument that the effective losses follow the effective plasma frequency *γ*_*m*_ = *ω*_*p*,*m*_*γ*_*Ag*_/*ω*_*p*,*Ag*_. We consider the best-case scenario, in which the silver losses remain constant 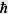
*γ*_*Ag*_ = 0.023 *eV*, independent of the patterning. In reality, they may additionally depend on the silver grain size, with a dependence of the form 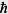
*γ*_*Ag*_ = 0.023 *eV* + const/*d*_*pattern*_, where *d*_*pattern*_ is a characteristic size of the patterning^47^.

## Additional Information

**How to cite this article**: Karalis, A. and Joannopoulos, J. D. ‘Squeezing’ near-field thermal emission for ultra-efficient high-power thermophotovoltaic conversion. *Sci. Rep.* **6**, 28472; doi: 10.1038/srep28472 (2016).

## Supplementary Material

Supplementary Information

## Figures and Tables

**Table 1:**
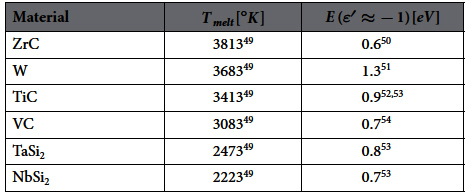
Plasmonic (metallic-type) refractory materials suitable for TPV emitter.

Listed are their high melting temperatures and the frequencies at which their real permittivity approximately equals −1 (using interpolation of the reference data) and thus they support a SPP mode in vacuum.
