# Supplementary material for: ‘Squeezing’ near-field thermal emission for ultra-efficient high-power thermophotovoltaic conversion
Source: Sci Rep. 2016 Jul 1;6:28472. doi: 10.1038/srep28472 (PMC4929479; doi:10.1038/srep28472)
Supplement: Supplementary Information [file srep28472-s1.pdf]

‘Squeezing’ near-field thermal emission for ultra-efficient high-power thermo-photovoltaic conversion

Aristeidis Karalis[1] and J. D. Joannopoulos[1,2]

SUPPLEMENTARY INFORMATION

We provide complete mathematical details for the definition and calculation of thermal transmissivity and emissivity for planar systems. We also provide additional modeling details, figures and discussion for the optimization of previously studied topologies and their comparison to currently proposed ones.

[1]Research Laboratory of Electronics, Massachusetts Institute of Technology, Cambridge, MA 02139, USA
[2]Department of Physics, Massachusetts Institute of Technology, Cambridge, MA 02139, USA
Correspondence: Aristeidis Karalis, Email: aristos@mit.edu, Phone: +1 (617) 253-6798

METHODS

Fluctuation-Dissipation Theorem (FDT)[1] and Poynting's Theorem (PT)[2]

Consider an isotropic object of relative dielectric permittivity $\varepsilon = \varepsilon' + \mathrm{i}\varepsilon''$, that can absorb photons, namely $\varepsilon'' > 0$ [for $\exp(-i\omega t)$ convention]. If it is brought at an absolute temperature T and optionally has a voltage V across it, FDT states that its thermally excited, randomly fluctuating atoms/molecules can be modeled as current sources $\mathbf{J}(\mathbf{r},\omega)$, with spatial correlation function

$$\langle J_\alpha(\omega,\mathbf{r}) J_\beta(\omega,\mathbf{r}')\rangle = \frac{\omega\varepsilon_o\varepsilon''(\omega,\mathbf{r})}{\pi}\hbar\omega\Theta_{VT}(\omega)\cdot\delta(\mathbf{r}-\mathbf{r}')\delta_{\alpha\beta} \qquad \text{(S1)}$$

where $\Theta_{VT}(\omega) = 1/\{\exp[(\hbar\omega - qV)/k_B T] - 1\}$ is the mean number of generated photons of frequency ω in thermo-chemical quasi-equilibrium[3] at voltage V and temperature T, ε_o is the permittivity of free space, $\hbar$ is the Planck constant divided by 2π, q is the electron charge, k_B is the Boltzmann constant, $\delta(\mathbf{r}' - \mathbf{r}'')$ is the Dirac delta function and $\delta_{\alpha\beta}$ is the Kronecker delta. In Θ, the additional vacuum-fluctuations term $1/2$ is omitted, as it does not affect the energy exchange between objects.

PT states energy conservation: the electromagnetic power absorbed inside a volume V is equal to that generated due to sources $\mathbf{J}$ in V minus that exiting the enclosing surface area A:

$$\int_V d\mathbf{r}\,\frac{\omega\varepsilon_o\varepsilon''}{2}|\mathbf{E}|^2 = \int_V d\mathbf{r}\cdot\frac{1}{2}\,\mathrm{Re}\{-\mathbf{E}\cdot\mathbf{J}^*\} - \oint_A d\mathbf{A}\cdot\frac{1}{2}\mathrm{Re}\{\mathbf{E}\times\mathbf{H}^*\} \qquad \text{(S2)}$$

Definition of thermal transmissivity for planar systems

The power absorbed by an object i, at $T_i = 0$, due to the thermal current-sources inside another object j, at $T_j \neq 0$ and with optionally $V_j \neq 0$ across it, is $P_{ij} = \int_0^\infty d\omega p_{ij}(\omega)/2\pi$, where the time-averaged power per unit frequency $p_{ij}(\omega)$ is identified in PT [Eq.(S2)] as

$$p_{ij}(\omega) = 4\cdot\frac{\omega\varepsilon_o}{2}\int_{V_i} d\mathbf{r}_i\,\varepsilon_i''(\omega,\mathbf{r}_i)\langle E_{i,\gamma}(\mathbf{r}_i)\cdot E_{i,\gamma}^*(\mathbf{r}_i)\rangle \qquad \text{(S3)}$$

A factor of 4 has been added in $p_{ij}(\omega)$, since only positive frequencies are considered in the Fourier decomposition of the time-dependent fields into frequency-dependent quantities[4]. The fields $\mathbf{E}_i(\mathbf{r}_i)$ and $\mathbf{H}_i(\mathbf{r}_i)$ due to the sources $\mathbf{J}_j(\mathbf{r}_j)$ can be calculated via the Green's functions $\mathbf{G}^{E,H}(\omega;\mathbf{r}_i,\mathbf{r}_j)$

$$\begin{bmatrix}\mathbf{E}_i(\mathbf{r}_i)\\ \mathbf{H}_i(\mathbf{r}_i)\end{bmatrix} = \int_{V_j} d\mathbf{r}_j\begin{bmatrix}\mathrm{i}\omega\mu_o\mathbf{G}^E(\omega;\mathbf{r}_i,\mathbf{r}_j)\\ \mathbf{G}^H(\omega;\mathbf{r}_i,\mathbf{r}_j)\end{bmatrix}\cdot\mathbf{J}_j(\mathbf{r}_j) \qquad \text{(S4)}$$

where μ_o is the magnetic permeability of free space. Using Eq.(S4), Eq.(S3) can be written

$$p_{ij}(\omega) = 4\cdot\frac{\omega\varepsilon_o(\omega\mu_o)^2}{2}\int_{V_i} d\mathbf{r}_i\,\varepsilon_i''(\omega,\mathbf{r}_i)\left\langle\int_{V_j} d\mathbf{r}_j\,G^E_{\gamma\alpha}(\mathbf{r}_i,\mathbf{r}_j)J_{j,\alpha}(\mathbf{r}_j)\int_{V_j} d\mathbf{r}_j'\,G^{E*}_{\beta\gamma}(\mathbf{r}_i,\mathbf{r}_j')J^*_{j,\beta}(\mathbf{r}_j')\right\rangle \qquad \text{(S5)}$$

Using the FDT from Eq.(S1),

$$p_{ij}(\omega) = \frac{\hbar\omega}{2\pi}\Theta_{V_jT_j}(\omega)\cdot 4k_o^4\int_{V_i} d\mathbf{r}_i\,\varepsilon_i''(\omega,\mathbf{r}_i)\int_{V_j} d\mathbf{r}_j\,\varepsilon_j''(\omega,\mathbf{r}_j)\,G^E_{\gamma\alpha}(\mathbf{r}_i,\mathbf{r}_j)G^{E*}_{\alpha\gamma}(\mathbf{r}_i,\mathbf{r}_j) \qquad \text{(S6)}$$

where $k_o = \omega\sqrt{\varepsilon_o\mu_o} = \omega/c$ is the wavevector of propagation and c the speed of light, in free space.

Consider now a planar system (uniform in xy) of N layers (stacked in z) of dielectric permittivities ε_n and thicknesses d_n. Then, $\mathbf{G}^{E,H}(\omega;\mathbf{r}_i,\mathbf{r}_j)$ can be written via their Fourier transforms

$$\mathbf{G}^{E,H}(\omega;\mathbf{r}_i,\mathbf{r}_j) = \iint \frac{dk_x dk_y}{(2\pi)^2}\mathbf{g}^{E,H}(\omega,k_{xy};z_i,z_j)e^{\mathrm{i}k_x x+\mathrm{i}k_y y} \tag{S7}$$

where $k_{xy} = \sqrt{k_x^2+k_y^2}$. Then in Eq.(S6), if k_x, k_y and k_x', k_y' are the integration variables for the two $\mathbf{G}^E$ instances, the integral $\int dx_j dy_j$ gives $\delta(k_x-k_x')\delta(k_y-k_y')$ and the integral $\int dx_i dy_i$ gives the (large) transverse system area A. Using also $\iint_{-\infty}^{+\infty} dk_x dk_y = 2\pi\int_0^\infty k_{xy}dk_{xy}$, the power *per unit area* becomes

$$\frac{P_{ij}}{A} = \int_0^\infty \frac{d\omega}{2\pi}\hbar\omega\Theta_{V_jT_j}(\omega)\, 4k_o^4 \int_{d_i} dz_i\, \varepsilon_i''(\omega,z_i)\int_{d_j} dz_j\, \varepsilon_j''(\omega,z_j)\int_0^\infty \frac{k_{xy}dk_{xy}}{2\pi} g^E_{\gamma\alpha}(z_i,z_j)g^{E*}_{\alpha\gamma}(z_i,z_j) \tag{S8}$$

We define the *thermal transmissivity* of photons from layer j to layer i, $\epsilon_{ij}(\omega,k_{xy})$, via the net power per unit area absorbed by i due to thermal sources in j, when $T_i = 0$:

$$\frac{P_{ij}}{A} = \int_0^\infty \frac{d\omega}{2\pi}\hbar\omega\Theta_{V_jT_j}(\omega)\int_0^\infty \frac{k_{xy}dk_{xy}}{2\pi}\epsilon_{ij}(\omega,k_{xy}) \tag{S9}$$

Then, from Eq.(S8) and (S9) we get

$$\boxed{\epsilon_{ij}(\omega,k_{xy}) = 4k_o^4\int_{d_j} dz_j \int_{d_i} dz_i\, \varepsilon_j''(\omega,z_j)\varepsilon_i''(\omega,z_i)g^E_{\gamma\alpha}(\omega,k_{xy};z_i,z_j)g^{E*}_{\alpha\gamma}(\omega,k_{xy};z_i,z_j)} \tag{S10}$$

The thermal transmissivity ϵ_{ij} is dimensionless ($\mathbf{g}$ has units of m) and it depends on the geometry and materials of the entire photonic system (via $\mathbf{g}$), but not on temperatures or voltages. Furthermore, when the system is reciprocal, $\mathbf{g}(z_i,z_j) = \mathbf{g}^T(z_j,z_i)$, so, from Eq.(S10), $\epsilon_{ij}(\omega,k_{xy}) = \epsilon_{ji}(\omega,k_{xy})$.

Calculation of thermal transmissivity for planar systems

To calculate $\epsilon_{ij}(\omega,k_{xy})$ via Eq.(S10) requires the evaluation of a difficult double integral. Instead, from PT [Eq.(S2)] and with $\varepsilon_{\alpha\beta\gamma}$ the Levi-Civita symbol for vector outer products, $p_{ij}(\omega)$ in Eq.(S3) is written

$$p_{ij}(\omega) = 4\cdot\int_{V_i} d\mathbf{r}_i\, \frac{1}{2}\mathrm{Re}\{-\langle E_{i,\alpha}J^*_{j,\alpha}\rangle\} - 4\cdot\varepsilon_{\alpha\beta\gamma}\oint_{A_i} dA_{i,\alpha}\frac{1}{2}\mathrm{Re}\{\langle E_{i,\beta}H^*_{i,\gamma}\rangle\} \tag{S11}$$

For thermal power transmission between *different* objects ($i \neq j$), the first term on the right-hand side of Eq.(S11) is zero. Using $\mathbf{G}^{E,H}$ from Eq.(S4), the second term becomes

$$p_{ij}(\omega) = -4\varepsilon_{\alpha\beta\gamma}\oint_{A_i} dA_{i,\alpha}\mathrm{Re}\left\{\frac{\mathrm{i}\omega\mu_o}{2}\langle\int_{V_j} d\mathbf{r}_j\, G^E_{\beta\mu}(\mathbf{r}_i,\mathbf{r}_j)J_{j,\mu}(\mathbf{r}_j)\int_{V_j} d\mathbf{r}_j'\, G^{H*}_{\gamma\nu}(\mathbf{r}_i,\mathbf{r}_j')J^*_{j,\nu}(\mathbf{r}_j')\rangle\right\} \tag{S12}$$

Using the FDT from Eq.(S1),

$$p_{ij}(\omega) = \frac{\hbar\omega}{2\pi}\Theta_{V_jT_j}(\omega)\cdot 4k_o^2\varepsilon_{\alpha\beta\gamma}\oint_{A_i} dA_{i,\alpha}\int_{V_j} d\mathbf{r}_j\, \varepsilon_j''(\omega,\mathbf{r}_j)\,\mathrm{Im}\{G^E_{\beta\mu}(\mathbf{r}_i,\mathbf{r}_j)G^{H*}_{\gamma\mu}(\mathbf{r}_i,\mathbf{r}_j)\} \tag{S13}$$

For a planar system, using Eq.(S7) and the definition Eq.(S9), ϵ_{ij} can be calculated by the single integral

$$\boxed{\epsilon_{ij}(\omega, k_{xy}) = 4k_o^2 \int_{d_j} dz_j \varepsilon_j''(\omega, z_j)\ \mathrm{Im}\left\{\varepsilon_{\beta\gamma} g^E_{\beta\mu}(\omega, k_{xy}; z_i, z_j) g^{H*}_{\gamma\mu}(\omega, k_{xy}; z_i, z_j)\Big|_{z_{i,min}}^{z_{i,max}}\right\}} \quad \text{(S14)}$$

where β, γ now run only through x, y in Cartesian coordinates or ρ, θ in cylindrical coordinates.

Eq.(S14) includes two evaluations at the boundaries of layer i. Layers 1 and N might be semi-infinite (unbounded) or bounded by a Perfect Electric Conductor (PEC) or a Perfect Magnetic Conductor (PMC). When $j < i$, the boundary term at $z_{i,max}$ exists, only if layer i is finite and there is another layer $> i$ with absorption or there is radiation at $z \to +\infty$ for mode (ω, k_{xy}). Similarly when $j > i$, for the term at $z_{i,min}$.

To calculate $\epsilon_{ij}(\omega, k_{xy})$ via Eq.(S14), one can construct a semi-analytical expression for the Green's functions $\mathbf{g}$, using a scattering matrix formalism, and then perform the z_j-integration analytically. We use the procedure outlined by Ref. 4, however, with the canonical scattering-matrix formulation[5]:

For a two-port with ports I and II, waves incoming to the ports have amplitudes a_I, a_{II} – for example, $(\mathbf{E}, \mathbf{H})_{I,in} = a_I(\boldsymbol{\phi}^E, \boldsymbol{\phi}^H)_I$ – and waves outgoing from the ports have amplitudes b_I, b_{II}. The wavefunctions ϕ^E, ϕ^H are normalized for unity complex power – for example, $\frac{1}{2}\mathbf{E}_{I,in} \times \mathbf{H}_{I,in} \cdot \hat{\mathbf{z}} = a_I^2 \leftrightarrow \frac{1}{2}\boldsymbol{\phi}_I^E \times \boldsymbol{\phi}_I^H \cdot \hat{\mathbf{z}} = 1$. Then, the scattering matrix is defined by $(b_I \quad b_{II})^T = \bar{\bar{\mathbf{S}}} \cdot (a_I \quad a_{II})^T$ and, with this definition, it is symmetric ($\bar{\bar{\mathbf{S}}} = \bar{\bar{\mathbf{S}}}^T$) for reciprocal systems and unitary ($\bar{\bar{\mathbf{S}}}^\dagger \bar{\bar{\mathbf{S}}} = \bar{\bar{\mathbf{I}}}$) for lossless systems.

At an interface of adjacent layers m, n, we use reflection and transmission coefficients for incidence from layer m: $r_{mn} = (X_m - X_n)/(X_m + X_n) = -r_{nm}$ and $t_{mn} = 2\sqrt{X_m X_n}/(X_m + X_n) = t_{nm}$, where the *admittance* of a TE wave in layer n is $X_n = k_{z,n}/\omega\varepsilon_n$ and the *impedance* of a TM wave is $X_n = k_{z,n}/\omega\mu_o$. Note that, with this convention, at $k_{xy} = 0$, although the TE and TM waves are identical, $r_{mn}^{TE} = -r_{mn}^{TM}$.

For the thermal system of layers, stacked from bottom to top, we define the amplitude coefficients (A_n, B_n, C_n, D_n) for the upward and downward propagating (or evanescent) waves in each layer[4] at the middle of the layer, except for a semi-infinite bottom layer 1 or top layer N, defined at their only interface. Let $\bar{\bar{\mathbf{S}}}_{1j}$ be the scattering matrix between layers 1, j [where $\bar{\bar{\mathbf{S}}}_{1j} = (0\ \ 1; 1\ \ 0)$, if $j = 1$ and semi-infinite], and $\bar{\bar{\mathbf{S}}}_{jN}$ between layers j, N. Also, let $r_1 = 0$, if the bottom boundary of layer 1 is open, $r_1 = -1$, if it is PEC for TE polarization or PMC for TM, and $r_1 = 1$, if it is PMC for TE or PEC for TM. Similarly, $r_N = 0$ or -1 or 1 for the top boundary of layer N. Then, the coefficients at the emitting layer j are:

$$S_{1,j} = \frac{\bar{\bar{\mathbf{S}}}_{1j}(1,2)\bar{\bar{\mathbf{S}}}_{1j}(2,1)r_1}{1 - \bar{\bar{\mathbf{S}}}_{1j}(1,1)r_1} + \bar{\bar{\mathbf{S}}}_{1j}(2,2)$$

$$S_{N,j} = \frac{\bar{\bar{\mathbf{S}}}_{jN}(2,1)\bar{\bar{\mathbf{S}}}_{jN}(1,2)r_N}{1 - \bar{\bar{\mathbf{S}}}_{jN}(2,2)r_N} + \bar{\bar{\mathbf{S}}}_{jN}(1,1) \quad \text{(S15)}$$

and
$$A_j = B_j S_{1,j} = C_j S_{N,j} = D_j = \frac{S_{1,j} S_{N,j}}{1 - S_{1,j} S_{N,j}} \quad \text{(S16)}$$

[Note: If layer $j = 1$ and semi-infinite ($r_1 = 0$), $S_{1,1} = 0 = A_1 = C_1 = D_1$ and $B_1 = S_{N,1}$.
If layer $j = N$ and semi-infinite ($r_N = 0$), $S_{N,N} = 0 = A_N = B_N = D_N$ and $C_N = S_{1,N}$.]

Now, let $\bar{\bar{\mathbf{S}}}_{ji}$ be the scattering matrix between the layers j, i, and assume that $j < i$. Then, the amplitude coefficients at layer i relative to those at layer j are:

$$
\begin{gathered}
B_{ij} = \frac{B_j - (1 + A_j)\bar{\bar{\mathbf{S}}}_{ji}(1,1)}{\bar{\bar{\mathbf{S}}}_{ji}(1,2)} \\
A_{ij} = (1 + A_j)\bar{\bar{\mathbf{S}}}_{ji}(2,1) + B_{ij}\bar{\bar{\mathbf{S}}}_{ji}(2,2) \\
D_{ij} = \frac{D_j - C_j\bar{\bar{\mathbf{S}}}_{ji}(1,1)}{\bar{\bar{\mathbf{S}}}_{ji}(1,2)} \\
C_{ij} = C_j\bar{\bar{\mathbf{S}}}_{ji}(2,1) + D_{ij}\bar{\bar{\mathbf{S}}}_{ji}(2,2)
\end{gathered}
\tag{S17}
$$

Let $x_n = X_n/|X_n| = x_n' + \mathrm{i}x_n''$, the complex z-wavevector $k_{z,n} = \sqrt{k_o^2\varepsilon_n - k_{xy}^2} = k_{z,n}' + \mathrm{i}k_{z,n}''$, the complex z-propagation phase $u_n = k_{z,n}d_n = u_{z,n}' + \mathrm{i}u_{z,n}''$, and $R_n = 2x_n'\sinh(u_{z,n}'')$, $I_n = 2x_n''\sin(u_{z,n}')$, if layer n is finite, or $R_n = x_n'$, $I_n = x_n''$, if it is semi-infinite. Then we finally calculate:

$$
\begin{aligned}
\epsilon_{ij}(\omega, k_{xy}) = R_j R_i &(A_{ij}A_{ij}^* + B_{ij}B_{ij}^* + C_{ij}C_{ij}^* + D_{ij}D_{ij}^*) \\
+ R_j I_i &(A_{ij}B_{ij}^* + B_{ij}A_{ij}^* + C_{ij}D_{ij}^* + D_{ij}C_{ij}^*) \\
+ I_j R_i &(A_{ij}C_{ij}^* + B_{ij}D_{ij}^* + C_{ij}A_{ij}^* + D_{ij}B_{ij}^*) \\
+ I_j I_i &(A_{ij}D_{ij}^* + B_{ij}C_{ij}^* + C_{ij}B_{ij}^* + D_{ij}A_{ij}^*)
\end{aligned}
\tag{S18}
$$

[Note: If layer $j = 1$ and semi-infinite, then $C_{i1} = D_{i1} = 0$.

If layer $i = N$ and semi-infinite *too*, then $A_{N1} = \bar{\bar{\mathbf{S}}}_{1N}(2,1)$, $B_{N1} = 0$, and $\epsilon_{N1} = x_1'x_N'\left|\bar{\bar{\mathbf{S}}}_{1N}(2,1)\right|^2$.]

Similar procedure can be followed, if $j > i$. Therefore, using Eq.(S15)-(S18), the thermal transmissivity $\epsilon_{ij}(\omega, k_{xy})$, as defined in Eq.(S9), can be fully calculated for planar systems in a semi-analytical way.

Definition and Calculation of thermal emissivity for planar systems

We also define the *thermal emissivity* of photons from layer j, $\epsilon_j(\omega, k_{xy})$, via the net power per unit area emitted outwards by j due to its thermal sources, when all other $T_{i\neq j} = 0$. Clearly, this power must be the sum of the powers transmitted to all other absorbing layers and the power potentially radiated into infinity, namely transmitted to the two semi-infinite boundary layers, so $\epsilon_j(\omega, k_{xy}) = \sum_{i\neq j}\epsilon_{ij}(\omega, k_{xy})$. This thermal emissivity is a generalization of the regular (k_{xy}-independent) emissivity[6]. Using Eq.(S14):

$$
\boxed{\epsilon_j(\omega, k_{xy}) = -4k_o^2\int_{d_j} dz_j\varepsilon_j''(\omega, z_j)\,\mathrm{Im}\left\{\varepsilon_{\beta\gamma}g_{\beta\mu}^E(\omega, k_{xy}; z_j', z_j)g_{\gamma\mu}^{H*}(\omega, k_{xy}; z_j', z_j)\Big|_{z_{j,min}}^{z_{j,max}}\right\}}
\tag{S19}
$$

With the same procedure, and $\tilde{R}_n = 2x_n'\exp(-u_{z,n}'')$, $\tilde{I}_n = 2x_n''\exp(-\mathrm{i}u_{z,n}')$, $\tilde{U}_n = 2[1 - \exp(-2u_{z,n}'')]$, if layer n is finite, or $\tilde{R}_n = 0$, $\tilde{I}_n = 2x_n''$, $\tilde{U}_n = 1$, if it is semi-infinite, we can also calculate:

$$
\begin{aligned}
\epsilon_j(\omega, k_{xy}) = x_j'^2\tilde{U}_j &+ R_j\{\tilde{R}_j\mathrm{Re}(A_j + D_j) + \mathrm{Im}[\tilde{I}_j(B_j + C_j)]\} \\
&+ I_j\{\tilde{R}_j\mathrm{Re}(B_j + C_j) + \mathrm{Im}[\tilde{I}_j(A_j + D_j)]\} - \mathrm{Eq.\,(S18)}|_{ij\to j}
\end{aligned}
\tag{S20}
$$

As explained in the main text, the transmissivity ϵ_{ij} and emissivity ϵ_j both have maximum value 1 for each of the two decoupled (for isotropic media TE/TM) polarizations.

Calculation of thermal self-transmissivity for planar systems

In some cases, we may want to calculate the power absorbed inside an object due to the thermal sources inside the *same* object, namely $i = j$. This may be useful to model thermal energy exchange between two different absorption mechanisms inside the same object, such as inter-band and free-carrier absorption in a semiconductor and the radiative recombination process, wherein photons absorbed by the former are re-emitted and then absorbed by the latter, e.g. increasing the saturation current of a pn-photodiode. For these cases, the first term F on the right-hand side of Eq.(S11) is non-zero. Using $\mathbf{G}^E$ from Eq.(S4) and using the FDT [Eq.(S1)] for the expectation value, we get

$$F = \frac{\hbar\omega}{2\pi}\Theta_{V_jT_j}(\omega)\cdot 4k_o^2\int_{V_j} d\mathbf{r}_j \varepsilon_j''(\omega,\mathbf{r}_j)\,\mathrm{Im}\left\{G_{\alpha\alpha}^E(\mathbf{r}_j,\mathbf{r}_j)\right\} \tag{S21}$$

Then, with a similar definition to Eq.(S9) for $i = j$, we arrive at the 'thermal form' of Poynting's Theorem [Eq.(S2)], for a layer j in a planar system:

$$\boxed{\epsilon_{jj}(\omega,k_{xy}) = 4k_o^2\int_{d_j} dz_j \varepsilon_j''(\omega,z_j)\,\mathrm{Im}\{g_{\alpha\alpha}^E(\omega,k_{xy};z_j,z_j)\} - \epsilon_j(\omega,k_{xy})} \tag{S22}$$

With $p = 1$ for TE waves or $p = (k_o^2 - k_{z,j}^2)/(k_o^2 + k_{z,j}^2)$ for TM waves, the integral term in Eq.(S22), denoted by $\epsilon_{\mathbf{J},j}(\omega,k_{xy})$, is calculated

$$\epsilon_{\mathbf{J},j}(\omega,k_{xy}) = 4\mathrm{Re}\left\{\frac{k_{z,j}'k_{z,j}''}{k_{z,j}^2}\left[(1+A_j+D_j)u_j + p(B_j+C_j)\sin(u_j)\right]\right\} \tag{S23}$$

Note that, for the term ϵ_{jj}, we are only interested in the (practical) case where layer j is finite ($d_j < \infty$), otherwise $\epsilon_{\mathbf{J},j}$ diverges. If a value is needed for a semi-infinite layer (for example, in Ref. 6), one can be provided by removing the divergent parts in Eq.(S23) and keeping only the structure-related terms:

If layer $j = 1$ and semi-infinite, $\epsilon_{\mathbf{J},1} = 4\mathrm{Re}\left\{k_{z,1}'k_{z,1}''/k_{z,1}^2\left[p\left(\bar{\bar{\mathbf{S}}}_{12}(1{,}1) - S_{N,1}\right)/2\mathrm{i}\right]\right\}$.

If layer $j = N$ and semi-infinite, $\epsilon_{\mathbf{J},N} = 4\mathrm{Re}\left\{k_{z,N}'k_{z,N}''/k_{z,N}^2\left[p\left(\bar{\bar{\mathbf{S}}}_{N-1,N}(2{,}2) - S_{1,N}\right)/2\mathrm{i}\right]\right\}$.

Superposition principle

In a system of multiple objects, each one at temperature T_j and with voltage V_j across it, the thermally-excited sources inside each object j generate photons with mean number $\Theta_{V_jT_j}(\omega)$. To find the net rate of photons R_i (or power P_i equivalently) emitted by object i, one must successively set to zero the temperatures of all objects but one each time, and then apply superposition. Using $\epsilon_i(\omega) = \sum_{j\neq i}\epsilon_{ji}(\omega)$,

$$R_i = \sum_j R_{ji} - R_{ij} = \int\frac{d\omega}{2\pi}\left\{\Theta_{V_iT_i}(\omega)\epsilon_i(\omega) - \sum_{j\neq i}\Theta_{V_jT_j}(\omega)\epsilon_{ij}(\omega)\right\} \tag{S24}$$

If the system is reciprocal, then $\epsilon_{ij}(\omega) = \epsilon_{ji}(\omega)$, so Eq.(S24) can also be written as

$$R_i = \int\frac{d\omega}{2\pi}\sum_j\left[\Theta_{V_iT_i}(\omega) - \Theta_{V_jT_j}(\omega)\right]\epsilon_{ji}(\omega) \tag{S25}$$

RESULTS

Surface-Plasmon-Polariton emitter and bulk semiconductor absorber[7,8]

Consider the structure in Figure S1a, which is the same as in Figure 1e, but with an additional 'base' region in the semiconductor, which has a very large (bulk) width and is more lightly doped than the thin front (pn-junction 'emitter') region of permittivity ε_a from Eq.(7). We use $d_{a,\text{base}} = 5\lambda_g$ and doping level $\omega_{p,a,\text{base}} = \omega_{p,a}/2 = 0.2\omega_g/\sqrt{\varepsilon_{\infty,a}}$, which would correspond, for example, to $N_e \approx 2 \times 10^{17} cm^{-3}$ electrons at $E_g = 0.4eV$ ($T_e = 1200°K$). Therefore, the permittivity in the 'base' region is taken as

$$\varepsilon_{a,\text{base}}(\omega) = 14 - \frac{0.04}{(\omega/\omega_g)^2 + \text{i}0.01\omega/\omega_g} + \text{i}0.7(\omega_g/\omega)\sqrt{14(\omega/\omega_g - 1)} \quad \text{(S26)}$$

We keep the silver back electrode, as it helps prevent radiation of photons within the semiconductor light-line[7]. We optimize again the efficiency vs load power, at $T_e = 1200°K$ with fixed $d_a = 0.07\lambda_g$ and at $T_e = 3000°K$ with fixed $d_a = 0.061\lambda_g$, to compare with the thin-film optimized structures at high power. The optimization parameters are $\omega_{p,e}/\omega_g$, d_{vac}/λ_g, qV/E_g.

The results are shown in Figure 3 with thin solid lines, cyan and orange for $T_e = 1200°K$ and $3000°K$ respectively. In Figure S1b-e are the TM emitter emissivities and emitter/load power densities for the two power levels P_1 and P_3 (shown with dots in Figure 3a) at $1200°K$.

At low power, clearly, the efficiency is significantly lower than in the thin-film absorber case (Figure 3a). Most of the losses come from free-carrier absorption within the semiconductor bulk (Figure 3b). This is because this single-mode impedance matching requires a much smaller vacuum gap at the same power level (Figure 3d) and thus the emitter SPP couples strongly with the absorber free carriers. At the same time, the bulk absorber geometry means that the semiconductor light-cone is filled with modes, also below bandgap, which also attribute to loss by free-carrier absorption (Figures S1b, S1d). This is more pronounced for smaller load power, where low-k_{xy} modes play a greater role. At the same power level P_1, the coupled-resonances structure has a much sharper power-density spectrum in Figure 1d than that of the single-resonance structure in Figure S1c, which also exhibits significant non-zero emission for all below-bandgap frequencies.

At higher power, the two structures behave similarly, as the thin-film is also non-resonant, so their power spectra at P_3 (Figures 4d, S1e) exhibit a strong emissivity peak, associated with the SPP mode at the interface of the doped semiconductor with the vacuum gap.

The distant silver back electrode does not impact efficiency greatly (Figure 3b). In previous studies with thick absorbers in both far-field[9] and near-field[7] TPV systems, a metallic back reflector has been shown to slightly improve efficiency by reflecting back into the emitter and recycling the undesired below-bandgap photons. However, this improvement was not significant and we see here that true removal of the radiation modes can only happen with a thin-film PV cell. Note that, for both the currently-proposed thin-film-absorber and also the bulk-absorber TPV systems, the thermalization losses (Figure 3b) stay relatively close to the T_a/T_e value dictated by the Carnot limit.

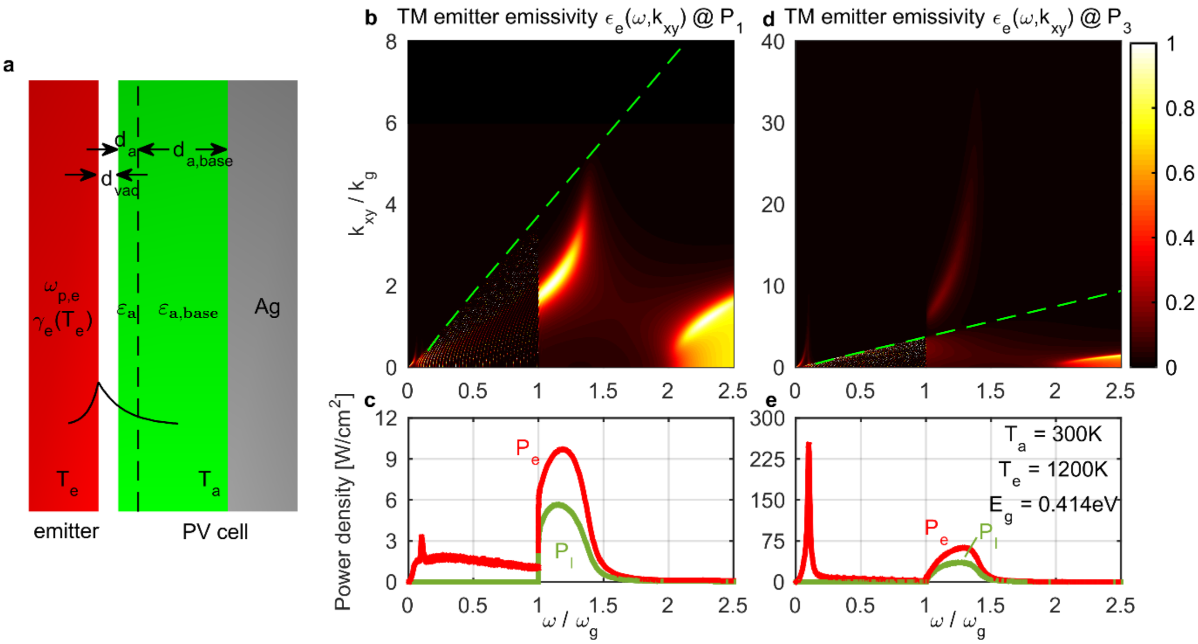

Supplementary Figure S1. (a) TPV structure of a plasmonic emitter and a silver-backed semiconductor bulk absorber. The semiconductor ε_a and $\varepsilon_{a,\text{base}}$ include free carriers to model the diffused-'emitter' front electrode and the doped 'base' respectively. The emitter-SPP modal energy profile is shown qualitatively. **(b,c,d,e)** Spectra for optimized results of Fig.3 cyan lines (at $T_e = 1200°K$, $T_a = 300°K$ and with $E_g = 4k_BT_e = 0.414eV$) at: **(b,c)** P_1, **(d,e)** P_3. **(b,d)** TM emitter emissivity $\epsilon_e(\omega, k_{xy})$ (color plot); green line is the semiconductor-material radiation cone. **(c,e)** TM emitter power $P_e(\omega)$ (red line) and load power $P_l(\omega)$ (green line) densities at the optimal-efficiency load voltage.

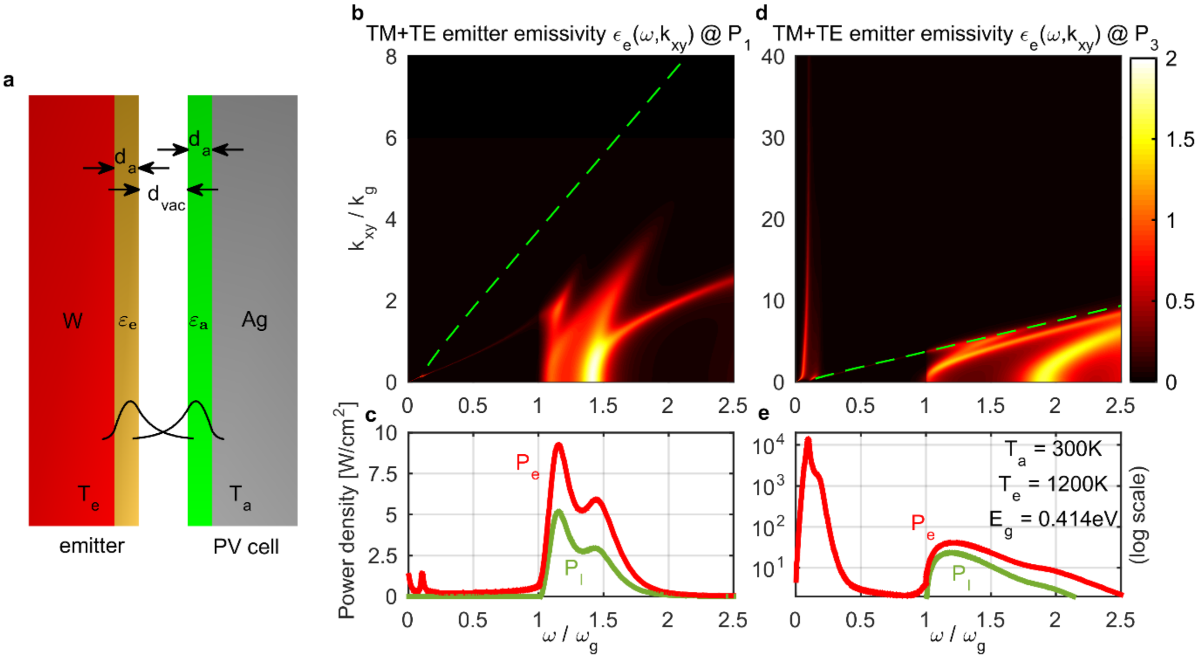

Supplementary Figure S2. (a) TPV structure of a tungsten-backed semiconductor thin-film emitter and a silver-backed semiconductor thin-film absorber. The semiconductor ε_e and ε_a include free carriers to model the elevated intrinsic carriers and the front electrode respectively. The coupled emitter- and absorber-waveguide modal energy profiles are shown qualitatively. **(b,c,d,e)** Spectra for optimized results of Fig.3 green lines (at $T_e = 1200°K$, $T_a = 300°K$ and with $E_g = 4k_BT_e = 0.414eV$) at: **(b,c)** P_1, **(d,e)** P_3. **(b,d)** Total (TM+TE) emitter emissivity $\epsilon_e(\omega, k_{xy})$ (color plot); green line is the semiconductor-material radiation cone. **(c,e)** Total emitter power $P_e(\omega)$ (red line) and load power $P_l(\omega)$ (green line) densities at the optimal-efficiency load voltage.

Thin-film semiconductor emitter and absorber[10]

Consider two coupled metal-backed semiconductor thin-films, shown in Figure S2a, where the bandgaps of the emitter and absorber semiconductors are matched. We perform the optimization at $T_e = 1200°K$, for comparison to the SPP-emitter results, although it is higher than most semiconductor melting temperatures (exceptions Ge: $\sim 1211°K$ and Si: $\sim 1638°K$). For the PV cell, we use the same semiconductor permittivity ε_a from Eq.(7) and a silver back electrode. For the emitter, we assume an undoped semiconductor with matched bandgap $\hbar\omega_g = 4k_B T_e = 0.414eV$ and permittivity ε_e of the same form of Eq.(7). However, at that close-to-melting temperature the thermally-excited intrinsic carriers will be extremely high and the mobility of those carriers greatly reduced. It is not easy to exactly predict those quantities, but based on estimations (using $n_i \sim T^{3/2} e^{-E_g/2k_B T}$ and $\mu \sim 1/T$ for n-GaAs[11]), we make the simple assumptions that carriers are 4 times higher than in the absorber, so $\sqrt{\varepsilon_{\infty,e}}\omega_{p,e} = 0.8\omega_g$, and that carrier mobility is 4 times lower, so $\gamma_e = 0.08\omega_g$. We also ignore the effect that the bandgap shifts to lower frequencies and gets smeared at high temperature (one could claim that an emitter semiconductor can be chosen, such that it has a high-energy bandgap at room temperature, which shifts down to meet the absorber at high temperature, although, in reality, this is likely possible only for few sets of semiconductor pairs). Finally, the emitter thin film is backed by tungsten W. By fitting the Drude model to room-temperature experimental data for tungsten in the range $(0.1 - 1)eV$, we get $\varepsilon_{\infty,W} = 36$, $\sqrt{\varepsilon_{\infty,W}}\hbar\omega_{p,W} = 5.88eV$ and $\hbar\gamma_W(300°K) = 0.058eV$; at $1200°K$, we increase the loss rate in proportion to the increase of the dc resistivity[12], so $\hbar\gamma_W(1200°K) = 0.33eV$. Since the real parts of ε_a, ε_e are quite similar and Ag, W will perform similarly for modal confinement, we take, for simplicity, equal thickness d_a of the two thin films, which is such that the first waveguide modes of the emitter and absorber couple just above bandgap. Our optimization parameters are thus d_{vac}/λ_g, d_a/λ_g and qV/E_g.

The results are shown again in Figure 3, with thin green lines, and in Figure S2b-e are the emitter emissivities (for both polarizations, so max value is 2) and emitter/load power densities for P_1 and P_3.

At lower power, efficiency is similarly high as the currently proposed (SPP-emitter) systems, as the benefits of the resonance with the thin-film absorber are present in this system too: high above-bandgap emissivity due to impedance-matched coupled resonances, removal of below-bandgap modes due to the metallic (Ag, W) reflectors (Figures S2b, S2c), and suppressed free-carrier absorption (Figure 3b) due to large vacuum gap (Figure 3d). Still, the SPP-emitter system exhibits a much narrower emissivity profile above the bandgap in comparison (Figures 1d vs. S2c).

As the desired load power increases, the necessary vacuum gap decreases (Figure 3d) and the 'good' waveguide modes meet their k_{xy}-limit, while emissivity related to the two (emitter and absorber) 'bad' free-carrier SPP (k_{xy}-unbounded) modes increases strongly (Figures S2d, S2e), thus free-carrier absorption dominates (Figure 3b) and efficiency drops very fast (Figure 3a). Note that, if these two SPP modes were at the same frequency (a resonance, which we intentionally avoided in these simulations), there would be strong coupling between them as well and the losses would be even greater.

It is certainly difficult to predict accurately the performance of this system, as accurate modeling of the semiconductors (especially at very high temperature) is non-trivial, but qualitatively one can always expect that there is a lower load-power limit for these systems.

REFERENCES

1. Rytov, S. M., Kravtsov, Y. A. & Tatarskii, V. I., *Principles of statistical radiophysics 3: elements of random fields* (Springer, Berlin, Heidelberg, New York, 1989).

2. Jackson, J. D., *Classical Electrodynamics*, 3rd ed. (Wiley, New York, 1999).

3. Wurfel, P., The chemical potential of radiation. *Journal of Physics C: Solid State Physics* **15**, 3967 (1982).

4. Francoeur, M., Mengüç, M. P. & Vaillon, R., Solution of near-field thermal radiation in one-dimensional layered media using dyadic Green's functions and the scattering matrix method. *Journal of Quantitative Spectroscopy & Radiative Transfer* **110**, 2002 (2009).

5. Haus, H. A., *Waves and fields in optoelectronics* (Prentice-Hall, Englewood Cliffs, NJ, 1984).

6. Karalis, A. & Joannopoulos, J. D., Temporal coupled-mode theory model for resonant near-field thermophotovoltaics. *Applied Physics Letters* **107** (4), 141108 (2015).

7. Bright, T. J., Wang, L. P. & Zhang, Z. M., Performance of Near-Field Thermophotovoltaic Cells Enhanced With a Backside Reflector. *Journal of Heat Transfer* **136**, 062701 (2014).

8. Ilic, O., Jablan, M., Joannopoulos, J. D., Celanovic, I. & Soljačić, M., Overcoming the black body limit in plasmonic and graphene near-field thermophotovoltaic systems. *Optics Express* **20** (S3), A366 (2012).

9. Charache, G. W. *et al.*, InGaAsSb thermophotovoltaic diode: Physics evaluation. *Journal of Applied Physics* **85** (4), 2247-2252 (1999).

10. Tong, J. K., Hsu, W.-C., Huang, Y., Boriskina, S. V. & Chen, G., Thin-film 'Thermal Well' Emitters and Absorbers for High-Efficiency Thermophotovoltaics. *Scientific Reports* **5**, 10661 (2015).

11. Sze, S. M., *Physics of Semiconductor Devices*, 2nd ed. (Wiley, New York, 1981).

12. Desal, P. D., Chu, T. K., James, H. M. & Ho, C. Y., Electrical Resistivity of Selected Elements. *Journal of Physical and Chemical Reference Data* **13** (4), 1069-1096 (1984).
